# Elucidating the Linkage Between Obesity‐Related Body Fat Indicators and Atrial Fibrillation: Supported by Evidence From Mendelian Randomization and Mediation Analyses

**DOI:** 10.1002/clc.70103

**Published:** 2025-03-05

**Authors:** Junxian Wang, Shengzhi Zhou, Xiaoming Xie, Wenlin Liu

**Affiliations:** ^1^ Department of General Medicine Affiliated Hospital of Guangdong Medical University Zhanjiang China; ^2^ Department of Cardiology Affiliated Hospital of Guangdong Medical University Zhanjiang China; ^3^ Department of Traditional Chinese Medicine Affiliated Hospital of Guangdong Medical University Zhanjiang China

**Keywords:** atrial fibrillation, body fat percentage, mediation analysis, Mendelian randomization study, trunk fat mass, whole body fat mass

## Abstract

**Objectives:**

To elucidating the linkage between obesity‐associated body fat indicators and atrial fibrillation (AF) using Mendelian Randomization (MR) and mediation analysis.

**Methods:**

The study utilized three independent genome‐wide association study (GWAS) datasets, with containing over 450 000 individuals each, to represent body fat indicators as the exposure variable. Additionally, two summary genetic datasets of AF were utilized as the clinical outcome. Single nucleotide polymorphisms (SNPs) with *p*‐values less than 5 × 10^−10^ were identified as instrumental variables (IVs) for MR analysis. The primary analysis method employed was the inverse‐variance weighting (IVW) model, supplemented by three additional models: MR‐Egger regression, weighted median, and maximum likelihood. Sensitivity analysis was conducted, encompassing tests for heterogeneity and horizontal pleiotropy, utilizing Cochran's Q, MR‐Egger intercept, and MR‐PRESSO tests to validate the reliability of the findings. Furthermore, a mediation analysis was conducted to explore potential mediators involved in the pathogenesis of AF.

**Results:**

The IVW model demonstrated that per 1‐SD increase in body fat indicators (body fat percentage, whole body fat mass, and trunk fat mass) is associated with an elevated risk of AF, with values of 63.1%, 55.0%, and 55.8% respectively. All three supplementary models arrived comparable conclusions with IVW model. The sensitivity analysis confirmed the absence of horizontal pleiotropy, thereby validating the reliability of the findings. Additionally, the mediation study indicates that hypertension and sleep apnea syndrome are identified as significant mediators during the pathogenesis of AF.

**Conclusions:**

The study reveals that individuals with a higher body fat percentage tend to exhibit a heightened genetic predisposition for susceptibility to AF. Meanwhile, hypertension and sleep apnea syndrome have been identified as key mediators contributing to the pathogenesis of AF.

**Abbreviations**
AF: Atrial fibrillation
CI: Confidence interval
GWAS: Genome‐wide association study
IV: Instrumental variable
IVW: Inverse variance weighting
MR: Mendelian randomization
OR: Odds ratio
SNP: Single‐nucleotide polymorphism

## 1 Introduction

Atrial fibrillation (AF) is a prevalent and potentially life‐threatening cardiac arrhythmia associated with acute ischemic stroke and heart failure. Based on a global epidemiological report, approximately 3 046 000 new cases of AF were diagnosed in 2017. Meanwhile, the global prevalence of AF in 2017 was 33% higher than the incidence reported in 1997, resulting in an estimated 37.574 million cases. Additionally, given the widespread aging population, the prevalence of AF is expected to increase, posing a significant challenge to global public health [1, 2].

Obesity, a well‐recognized independent risk factor for cardiovascular and metabolic diseases, affects approximately 30% of the world's population. Obesity typically arises from sustained accumulation of body fat and is categorized by a body mass index (BMI) of 30 kg/m^2^ or above, according to the World Health Organization's criteria. Nevertheless, the obese phenotype exhibits diverse characteristics and is not exclusively determined by BMI, rather, it encompasses measurements of body fat distribution, including leg fat, liver fat, and skeletal muscle mass. Detailed characterization of individual body components enhances our comprehension of metabolic, endocrine, and genetic profiles linked to obesity and its related metabolic risks [3, 4, 5].

Based on the global burden database, researchers observed an escalation in the occurrence of obesity, resulting in an estimated 4 million fatalities in 2015, with over two‐thirds attributed to cardiovascular disease [6]. Observational studies also demonstrated that obese/overweight individuals exhibit higher incidences, prevalence, severity, and rate of progression of AF compared to those with a normal weight [7, 8, 9]. However, observational studies inherently have limitations, which encompass reverse causation, measurement error, and potential bias, that hinder clarification of the association between obesity and AF, particularly the potential causal link, remaining enigmatic. Hence, innovative and rigorous research approaches aimed at minimizing these biases are crucial.

Mendelian randomization (MR) is a statistical technique widely used in epidemiological and genetic studies to elucidate causal relationships between exposure factors and outcomes. MR is rooted in Mendel's law of inheritance, which outlines how genetic variants undergo random allocation during meiosis [10]. MR employs instrumental variables, particularly genetic variations like single nucleotide polymorphisms (SNPs) associated with a specific risk factor (e.g., obesity‐associated body fat indicators), to investigate whether the chosen risk factor has a causal influence on the outcome of interest (e.g., AF). In the absence of randomized controlled trials (RCTs), MR studies serve as an alternative approach that mimics the RCT process due to the random allocation of genetic variants during meiosis. Consequently, MR offers advantages over traditional observational studies by minimizing confounding risks and clarifying reverse causality, making it a powerful tool for exploring causality in epidemiological research [11].

We aim to employ extensive Genome‐Wide Association Study (GWAS) datasets in this study, to identify a potential causal association between obesity‐associated body fat indicators and AF through MR and mediation analysis. This approach will yield robust evidence regarding the impact of obesity on the etiology of AF.

## 2 Materials and Methods

### 2.1 MR Study Design and Information of GWAS Datasets

To guarantee unbiased findings in this MR study, three fundamental assumptions were established. Firstly, the genetic instrumental variables (IVs) chosen from among the single nucleotide polymorphisms (SNPs), must exhibit a strong association with the exposure factor. Secondly, the IVs must remain independent of any potential confounders that could potentially correlate with both the exposure factors and outcomes. Lastly, the influence of the IVs on the outcome is solely mediated by the exposure factor [12].

To gain a deeper understanding of the causal relationship between obesity‐associated body fat indicators (exposure) and atrial fibrillation (outcome), it is imperative to select the most recent GWAS datasets, encompassing the largest cohort of participants and an adequate number of sequenced SNPs, for MR analysis. Obesity‐associated body fat indicators were represented by three independent GWAS datasets, namely body fat percentage, whole body fat mass, and trunk fat mass. Each of these datasets encompassed over 450 000 individuals. The three GWAS datasets were sourced from the UK‐Biobank database, a comprehensive longitudinal study meticulously designed to provide detailed genetic and health information on European populations [13]. The bioelectrical impedance technique, a practical approach, was utilized to measure these indices, enabling the estimation of body fat [14]. A low, safe electrical current is administered to the body, allowing for the assessment of its composition and subsequent determination of body fat percentage. The Tanita BC418MA body composition analyzer provided the reported results. AF is defined as a chaotic, rapid (300–500 beats per minute), and irregular atrial rhythm. It was common in clinical usually diagnosed by electronic cardiogram or Holter [15]. In our study, two independent GWAS datasets associated with AF were contributed by Jonas B. Nielsen et al [16]. and Carolina Roselli et al. [17], including 60 620 and 65 446 cases (both paroxysmal and permanent atrial fibrillation) respectively. The GWAS datasets for four mediators, namely coronary atherosclerosis, hypertension, Type 2 diabetes, and sleep apnea syndrome, originated from the FinnGen database [18]. To mitigate potential biases stemming from sample overlap, distinct consortiums were sourced for GWAS datasets related to exposure, mediators, and outcomes. Furthermore, the GWAS datasets primarily comprised individuals of European descent, thereby mitigating biases associated with population stratification. Detailed information on the datasets used in the study is listed in Table 1. As all the GWAS datasets involved in the study were shared in the IEU GWAS (https://gwas.mrcieu.ac.uk/datasets/), a publicly accessible GWAS summary database [19], the ethical committee approval requirement was waived.

**Table 1:** Basic information about the GWAS datasets used in the study.

Traits	GWAS ID	Years	Population	Sample size
**Body fat indicators**				**Total sample**
Body fat percentage	ukb‐b‐8909	2018	European	454 633
Whole body fat mass	ukb‐b‐19393	2018	European	454 137
Trunk fat mass	ukb‐b‐20044	2018	European	454 588
**Atrial fibrillation**				**Case/Control**
Atrial fibrillation	ebi‐a‐GCST006414	2018	European	60 620/970 216
	ebi‐a‐GCST006061	2018	European	55 114/482 295
**Mediators**				**Case/Control**
Coronary atherosclerosis	finn‐b‐I9_CORATHER	2021	European	23 363/187 840
Hypertension	finn‐b‐I9_HYPERTENSION	2021	European	55 955/162 837
Type 2 diabetes	finn‐b‐E4_DM2	2021	European	32 469/183 185
Sleep apnea syndrome	finn‐b‐G6_SLEEPAPNO	2021	European	16 761/201 194

### 2.2 Selection Criteria for SNPs Used as Instrumental Variables

SNPs should be independently associated with the obesity‐associated body fat indicators were extracted and treated as instrumental variables according to the following conditions: (1) a genome‐wide significance threshold settled as *p*‐value < 5 × 10^−10^, (2) independence among SNPs in linkage disequilibrium (*r* ^2^ < 0.0001; clumping distance, 100 000 kb), (3) The independence of SNPs was ensured using the PhenoScanner database (http://www.phenoscanner.medschl.cam.ac.uk/) to identify and remove SNPs that are associated with potential confounders or AF, (4) F‐statistics exceeding 10 were utilized to assess the robustness of the instrumental variables in mitigating instrumental bias. The SNPs serving as instrumental variables were aligned with those in AF‐associated GWAS datasets to establish genetic associations. The SNP‐phenotype and SNP‐outcome summary statistics were standardized to ensure effect size alignment, excluding palindromic SNP [20].

### 2.3 Mendelian Randomization Study and Sensitivity Analysis

The Mendelian randomization study employed the random effect inverse‐variance weighting (IVW) model as the primary analysis method [21] to assess the potential causal relationship between the exposure and AF across various scenarios. Moreover, three additional models, namely MR‐Egger regression [22], weighted median [23], and maximum likelihood [24] respectively were applied to complement the conclusion of IVW model. The evidential threshold for MR analysis was set at a *p*‐value of less than 0.017 (0.05/3), derived from the Bonferroni correction method. A *p*‐value ranging from the Bonferroni‐corrected evidential threshold to 0.05 was considered indicative of a potential association [25].

Sensitivity analysis was performed to evaluate the reliability and stability of the MR findings. It encompassed three key components as follows: (1) Cochran's Q test is used to identified heterogeneity based on IVW model and MR‐Egger regression model. (2) The horizontal pleiotropy test was conducted using the MR‐Egger intercept [26] and MR‐PRESSO test [27]. A *p*‐value less than 0.05 was considered statistically significant in both the Cochran's Q test and the horizontal pleiotropy test. (3) The “leave‐one‐out” test involves successively excluding each SNP and repeating the IVW analysis to determine if the estimated causal relationship is influenced by a particular SNP.

We reported the MR effects as odds ratios (OR) accompanied by corresponding 95% confidence intervals (CI) and *p*‐values. The R 4.0.3 software, along with the “TwoSampleMR” [28] and “MR‐PRESSO” [27] packages, was utilized for data processing and visualization of the results.

### 2.4 Mediation Analysis

**Figure 1:**
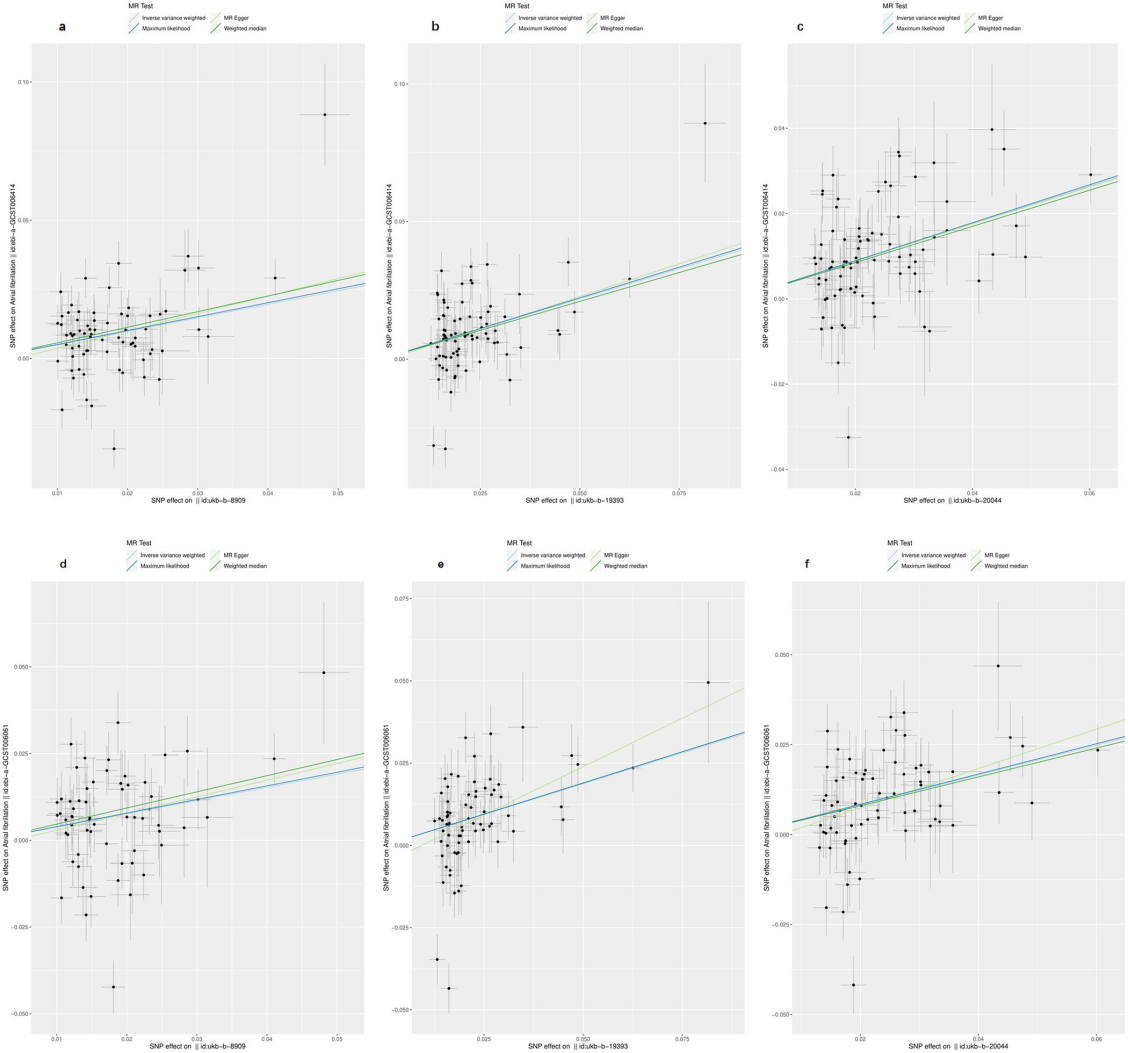
Scatter plots of the Mendelian randomization study results. Note: Each scatter plot point is an instrumental variable SNP. Each diagonal line in a different color is a testing model. The figure‐a, figure‐b, figure‐c represents the relationship of obesity‐associated body fat indicators “Body fat percentage,” “Whole body fat mass,” “Trunk fat mass” and AF in ebi‐a‐GCST006414 GWAS data set. The figure‐d, figure‐e, figure‐f represents the relationship of obesity‐associated body fat indicators “Body fat percentage,” “Whole body fat mass,” “Trunk fat mass” and AF in ebi‐a‐GCST006061 GWAS data set.

Mediation analysis aims to clarify the mechanisms by which a particular exposure affects an outcome [29]. We utilized a two‐stage MR methodology to investigate whether the impact of obesity‐associated body fat indicators on the risk of AF is mediated by four interested diseases (coronary atherosclerosis, hypertension, Type 2 diabetes, and sleep apnea syndrome). In the first stage, we estimated the relationship between each exposure. In the second stage, we identified the association between each mediator disease and AF [20]. To determine the mediating effect, we multiplied the effect value obtained in the first step by the effect value in the second step. We conducted a mediation analysis employing the IVW model, and considered a *p*‐value of less than 0.05 as statistically significant. Please refer to the Supporting Information S1: Figure 1 for detailed illustrations of the mediation analysis process.

## 3 Results

### 3.1 Instrumental Variables Identified and Results of Mendelian Randomization

A total of 72, 80, and 82 SNPs were ultimately identified as IVs from different obesity ‐proxied phenotype (body fat percentage, whole body fat mass, and trunk fat mass, respectively) in ebi‐a‐GCST006414 AF‐association GWAS datasets; while 62, 72, and 71 were identified, respectively, in ebi‐a‐GCST006061 AF‐association GWAS. The F‐statistic scores of the selected SNPs were ranged from 50 to 120, indicating a low risk of weak‐instrument bias.

Based on the findings from the random effect IVW model as the primary criterion, a causal relationship between all the three obesity‐proxied phenotypes (body fat percentage, whole body fat mass and trunk fat mass) and AF could be inferred in both AF‐associated GWAS datasets. Moreover, the conclusion was also supported by the rest three supplement models. Detailed results of MR study are displayed in Table 2 and is illustrated as a scatter plot (Figure 1).

**Table 2:** Results of the Mendelian randomization study.

	Atrial fibrillation (ebi‐a‐GCST006414)				Atrial fibrillation (ebi‐a‐GCST006061)			
	SNPs (n)	beta	OR (95%CI)	*p*‐value	SNPs (n)	beta	OR (95%CI)	*p*‐value
**Body fat percentage**								
Inverse variance weighted	72	0.489	1.631 (1.392–1.911)	< 0.001*	62	0.382	1.465 (1.217–1.763)	< 0.001*
MR Egger regression	72	0.631	1.879 (1.173–3.009)	0.011*	62	0.480	1.616 (0.936–2.790)	0.089
Weighted median	72	0.564	1.758 (1.494–2.068)	< 0.001*	62	0.466	1.593 (1.319–1.923)	< 0.001*
Maximum likelihood	72	0.504	1.655 (1.497–1.829)	< 0.001*	62	0.392	1.480 (1.320–1.660)	< 0.001*
**Whole body fat mass**								
Inverse variance weighted	80	0.438	1.550 (1.382–1.737)	< 0.001*	72	0.374	1.453 (1.285–1.645)	< 0.001*
MR Egger regression	80	0.473	1.605 (1.197–2.153)	0.002*	72	0.588	1.800 (1.321–2.454)	< 0.001*
Weighted median	80	0.419	1.521 (1.345–1.720)	< 0.001*	72	0.379	1.461 (1.288–1.658)	< 0.001*
Maximum likelihood	80	0.445	1.561 (1.454–1.676)	< 0.001*	72	0.379	1.461 (1.348–1.583)	< 0.001*
**Trunk fat mass**								
Inverse variance weighted	82	0.441	1.555 (1.409–1.715)	< 0.001*	71	0.414	1.514 (1.343–1.706)	< 0.001*
MR Egger regression	82	0.434	1.544 (1.186–2.010)	0.002*	71	0.548	1.730 (1.264–2.369)	0.001*
Weighted median	82	0.424	1.529 (1.360–1.719)	< 0.001*	71	0.401	1.494 (1.308–1.707)	< 0.001*
Maximum likelihood	82	0.446	1.562 (1.458–1.673)	< 0.001*	71	0.421	1.524 (1.408–1.649)	< 0.001*

*Note:* SNPs means number of SNP as instrumental variables. Beta means effect value. * indicates that the results have statistical differences.

Abbreviations: CI, Confidence Interval; OR, Odds Ratio.

### 3.2 Results of Sensitivity Analysis

The Cochran's Q test results, utilizing the IVW model and MR‐Egger regression, demonstrated the presence of heterogeneity (*p* < 0.05) among instrumental variables within obesity‐associated body fat exposures. To address the heterogeneity in the MR study, we primarily relied on the random‐effects IVW model. Additionally, the MR‐Egger intercept and MR‐PRESSO test results both confirmed that the instrumental variables for each obesity‐related body fat indicator passed the horizontal multiplicity test (Table 3). Furthermore, the “leave‐one‐out” method results (Supporting Information S1: Figure 2) revealed that no individual SNP had a significant impact on the overall outcome. In summary, the sensitivity analysis findings support the reliability of the MR conclusions.

**Table 3:** The results of the heterogeneity and horizontal pleiotropy tests.

Exposures	AF‐related GWAS data set	Heterogeneity test (Cochran Q test)		Horizontal pleiotropy test	
		MR‐Egger regression	IVW model	MR‐Egger intercept	MR‐PRESSO test
Body fat percentage	ebi‐a‐GCST006414	< 0.001	< 0.001	0.533	0.669
	ebi‐a‐GCST006061	< 0.001	< 0.001	0.708	0.428
Whole body fat mass	ebi‐a‐GCST006414	< 0.001	< 0.001	0.798	0.931
	ebi‐a‐GCST006061	< 0.001	< 0.001	0.145	0.493
Trunk fat mass	ebi‐a‐GCST006414	< 0.001	< 0.001	0.957	0.807
	ebi‐a‐GCST006061	< 0.001	< 0.001	0.370	0.469

*Note:* A *p*‐value less than 0.05 was deemed statistically significant in both the Heterogeneity and Horizontal Pleiotropy tests.

### 3.3 Results of Mediation Analysis

The results of the mediation analysis identified hypertension and sleep apnea syndrome as significant mediators linking obesity and AF pathogenesis. Analyzing three obesity‐associated body fat indicators—body fat percentage, whole body fat mass, and trunk fat mass—the study found hypertension to have mediation effects of 0.094 (95% CI: 0.060–0.134), 0.072 (95% CI: 0.046–0.102), and 0.059 (95% CI: 0.037–0.084), corresponding to mediation proportions of 19.22%, 16.44%, and 13.38% respectively, in AF‐related GWAS data set ebi‐a‐GCST006414. Additionally, hypertension demonstrated mediation effects of 0.092 (95% CI: 0.057–0.133), 0.071 (95% CI: 0.045–0.100), and 0.058 (95% CI: 0.034–0.084) respectively. Correspondingly, the mediation proportions in ebi‐a‐GCST006061 AF‐related GWAS data set were 24.08%, 18.98%, and 14.01% respectively.

The mediation effect of sleep apnea syndrome on three obesity‐associated body fat indicators (Body fat percentage, Whole body fat mass, and Trunk fat mass), was 0.120 (95% CI: 0.047–0.199), 0.099 (95% CI: 0.040–0.162) and 0.089 (95% CI: 0.034–0.147) respectively. The corresponding proportion of mediation effect in AF‐related GWAS data set ebi‐a‐GCST006414was account for 24.54%, 22.60%, and 20.18% respectively.

Additionally, sleep apnea syndrome demonstrated mediation effects of 0.100 (95% CI: 0.006–0.199), 0.082 (95% CI: 0.005–0.161) and 0.074 (95% CI: 0.005–0.147) respectively. Correspondingly, the mediation proportions in ebi‐a‐GCST006061 AF‐related GWAS data set were 26.18%, 21.93%, and 17.87% respectively. The detailed results of mediation analysis were demonstrated in Table 4.

**Table 4:** The results of the mediation analysis.

Exposures	Mediators	Outcomes			
		Atrial fibrillation (ebi‐a‐GCST006414)		Atrial fibrillation (ebi‐a‐GCST006061)	
		Mediation effect	Proportion of Mediation effect	Mediation effect	Proportion of Mediation effect
Body fat percentage (ukb‐b‐8909)	Coronary atherosclerosis	0.015 (0.004–0.030)	3.07%	0.011 (0.0002–0.026)	2.88%
	Hypertension	0.094 (0.060–0.134)	19.22%	0.092 (0.057–0.133)	24.08%
	Type 2 diabetes	NA	NA	NA	NA
	Sleep apnea syndrome	0.120 (0.047–0.199)	24.54%	0.100 (0.006–0.199)	26.18%
Whole body fat mass (ukb‐b‐19393)	Coronary atherosclerosis	0.012 (0.004–0.023)	2.74%	0.009 (0.001–0.020)	2.41%
	Hypertension	0.072 (0.046–0.102)	16.44%	0.071 (0.045–0.100)	18.98%
	Type 2 diabetes	NA	NA	NA	NA
	Sleep apnea syndrome	0.099 (0.040–0.162)	22.60%	0.082 (0.005–0.161)	21.93%
Trunk fat mass (ukb‐b‐20044)	Coronary atherosclerosis	0.007 (0.001–0.016)	1.59%	NA	NA
	Hypertension	0.059 (0.037–0.084)	13.38%	0.058 (0.034–0.084)	14.01%
	Type 2 diabetes	NA	NA	NA	NA
	Sleep apnea syndrome	0.089 (0.034–0.147)	20.18%	0.074 (0.005–0.147)	17.87%

*Note:* The mediation effect refers to the quantitative impact of each mediator on the outcome. The proportion of the mediated effect can be determined by dividing the mediated effect by the total effect.

## 4 Discussion

Obesity has been implicated as a risk factor for the increased occurrence of AF. However, prior research has predominantly focused on BMI analysis, overlooking the role of adipose tissue distribution and function in disease severity [3, 30]. To date, assessments of adiposity indices beyond BMI, specifically related to AF risk, are still limited, and the relationship between body fat distribution and AF development remains unclear. In the research, we utilized the Mendelian randomization and mediation analyses, with the latest and most comprehensive GWAS data from European cohorts to further explore the scientific problem. The study is expected to provide insights into the understanding of obesity and AF, surpassing the limitations of BMI alone.

The IVW model showed that a 1‐SD increase in obesity‐related body fat indicators (body fat percentage, whole body fat mass, and trunk fat mass) is linked to an elevated risk of AF, with values of 63.1%, 55.0%, and 55.8% respectively. The three supplementary models (MR‐Egger regression, weighted median, and maximum likelihood) consistently concurred with the outcomes of the IVW model. The sensitivity analysis affirmed the absence of horizontal pleiotropy, thereby confirming the stability and reliability of our conclusions. Moreover, the mediation study implies that hypertension and sleep apnea syndrome could play significant roles as mediators in the development of AF.

In the Rotterdam Study, a population‐based, prospective investigation, Maryam Kavousi and colleagues observed a significant dose–response relationship between body fat accumulation and AF. Their findings suggest that various body fat depots are associated with the onset of AF [31]. Consistent with our findings, a meta‐analysis reported both body fat percentage and body fat mass were regarded as risk factors of AF. A 5 kg increase in body fat mass was associated with a 9% increased risk of AF (95% CI: 1.02–1.16), while a 10% increase in body fat percentage was associated with a 10% increased risk of AF (95% CI: 0.92–1.33) [32]. A Swedish cohort study, led by Isac Zia et al., involving 25 961 individuals, demonstrated that a 10% increase in body fat percentage is associated with a 21% increased risk of AF in men and a 45% increased risk in women [33]. Likewise, a prospective study encompassing 55 273 Danish individuals confirmed the link between higher body fat at any measured location and an increased risk of AF. The adjusted hazard ratio (HR) for every 1 sex‐specific standard deviation (SD) increase in body fat mass was 1.29 (95% confidence interval [CI], 1.24–1.33) [34]. Meanwhile, comparable results have been reported in Asian populations. Ho Geol Woo et al. conducted a study with a median follow‐up of 9.5 years (interquartile range: 9.2–10.1) and reported that higher body fat mass was associated with increased risks of AF, with hazard ratios of 1.345 (95% confidence interval: 1.221–1.483) for men in the fifth quintile and 1.420 (95% confidence interval: 1.274–1.591) for women in the fifth quintile [35].

On the contrary, numerous studies have consistently shown that effective obesity management can attenuate the natural progression of AF. These clinical trial outcomes strongly support the hypothesis that excessive body fat accumulation may exacerbate the severity of AF [36, 37, 38, 39].

Obesity, often resulting from disrupted body fat metabolism or accumulation, frequently coincides with diabetes, hypertension, and obstructive sleep apnea. The mediation analysis identified hypertension and sleep apnea syndrome as key mediators in the development of AF. Magnani et al. offer a plausible mechanistic explanation for the interplay between obesity and these mediators, ultimately leading to AF. Obesity, influenced by metabolic factors like hypertension, dyslipidemia, and insulin resistance, alongside mechanical effects such as obstructive sleep apnea (OSA), increased intrathoracic pressure, coronary disease, ventricular hypertrophy, and inflammation, ultimately results in atrial fibrillation (AF) through atrial adaptation marked by elevated atrial pressures, enlargement, and altered electrical function [40].

Obesity increases the risk of AF through various mechanisms, including structural and electrical remodeling, thereby contributing to the formation of an arrhythmogenic substrate. Experimental studies utilizing the ovine model have demonstrated that short‐term weight gain results in progressive atrial remodeling. This remodeling involves increased deposition of fibrous tissue, upregulated expression of endothelin receptors, and altered atrial conduction, ultimately resulting in a higher susceptibility to AF induction [41]. Meanwhile, Mahajan et al. partially investigated the influence of weight loss on the progression of AF. Among obese sheep, weight loss was associated with a decrease in both inflammatory and fibrotic markers [42].

It is important to acknowledge several limitations in this study. Firstly, the heterogeneity test results indicated the presence of variance among the instrumental variables within the study, Despite utilizing the random effects IVW model to minimize heterogeneity in the MR study. Secondly, the GWAS datasets primarily centered on the European population, indicating that the findings might not be universally applicable across other ethnicities. Additionally, there is a need for further exploration of the biological functions of SNPs as instrumental variables and their role in aggravating AF.

## 5 Conclusion

The study reveals that individuals with a higher body fat percentage tend to exhibit a heightened genetic predisposition for susceptibility to AF. Additionally, hypertension and sleep apnea syndrome have been identified as key mediators contributing to the pathogenesis of AF. The findings have potential implications for AF prevention.

## Author Contributions

Junxian Wang contributed to the conceptualization and writing of the article, while Shengzhi Zhou and Xiaoming Xie were tasked with data mining and analysis. Wenlin Liu contributed to the conceptualization and writing of the article, while also overseeing scientific supervision. All authors participated in the review and approval of the final manuscript.

## Ethics Statement

The requirement of ethical approval was waived for the studies involving humans because the variable genetic information involved in this study was extracted from the Integrative Epidemiology Unit (IEU) GWAS database (https://gwas.mrcieu.ac.uk/), which is a publicly available GW AS summary database. The studies were conducted in accordance with the local legislation and institutional requirements. The participants provided their written informed consent to participate in these studies.

## Consent

The authors have nothing to report.

## Conflicts of Interest

The authors declare no conflicts of interest.

## Supporting information

Supporting information.

## Data Availability

The data that support the findings of this study are available on request from the corresponding author. The data are not publicly available due to privacy or ethical restrictions. Genetic variable information on SNPs was retrieved from the publicly accessible IEU GWAS database located at https://gwas.mrcieu.ac.uk/datasets/.
