# Supplementary material for: Elucidating the Linkage Between Obesity‐Related Body Fat Indicators and Atrial Fibrillation: Supported by Evidence From Mendelian Randomization and Mediation Analyses
Source: Clin Cardiol. 2025 Mar 5;48(3):e70103. doi: 10.1002/clc.70103 (PMC11882476; doi:10.1002/clc.70103)
Supplement: Supplementary file 1 — Supporting information. [file CLC-48-e70103-s001.docx]

**Supplementary Figure-1 The flowchart of mediation analysis**


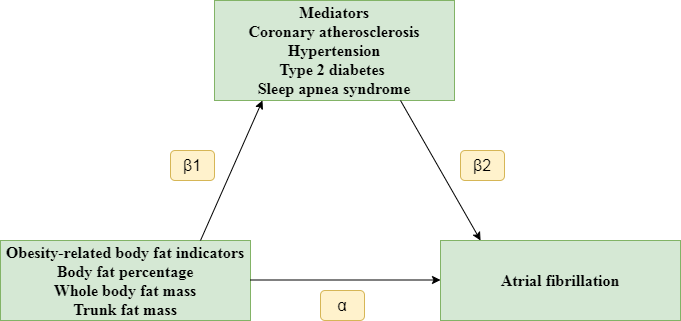


**Note:** This figure illustrates the direct and mediating effects of obesity-associated body fat indicators (exposure) on the risk of AF. Here, α denotes the direct effect of exposure on AF, while β1β2 represents the mediating effect of each indicators on AF through the included mediators respectively. The total effect is captured by the sum of α+β1β2.

**Supplementary Figure-2 Results of the “leave-one-out” method in the sensitivity analysis.**


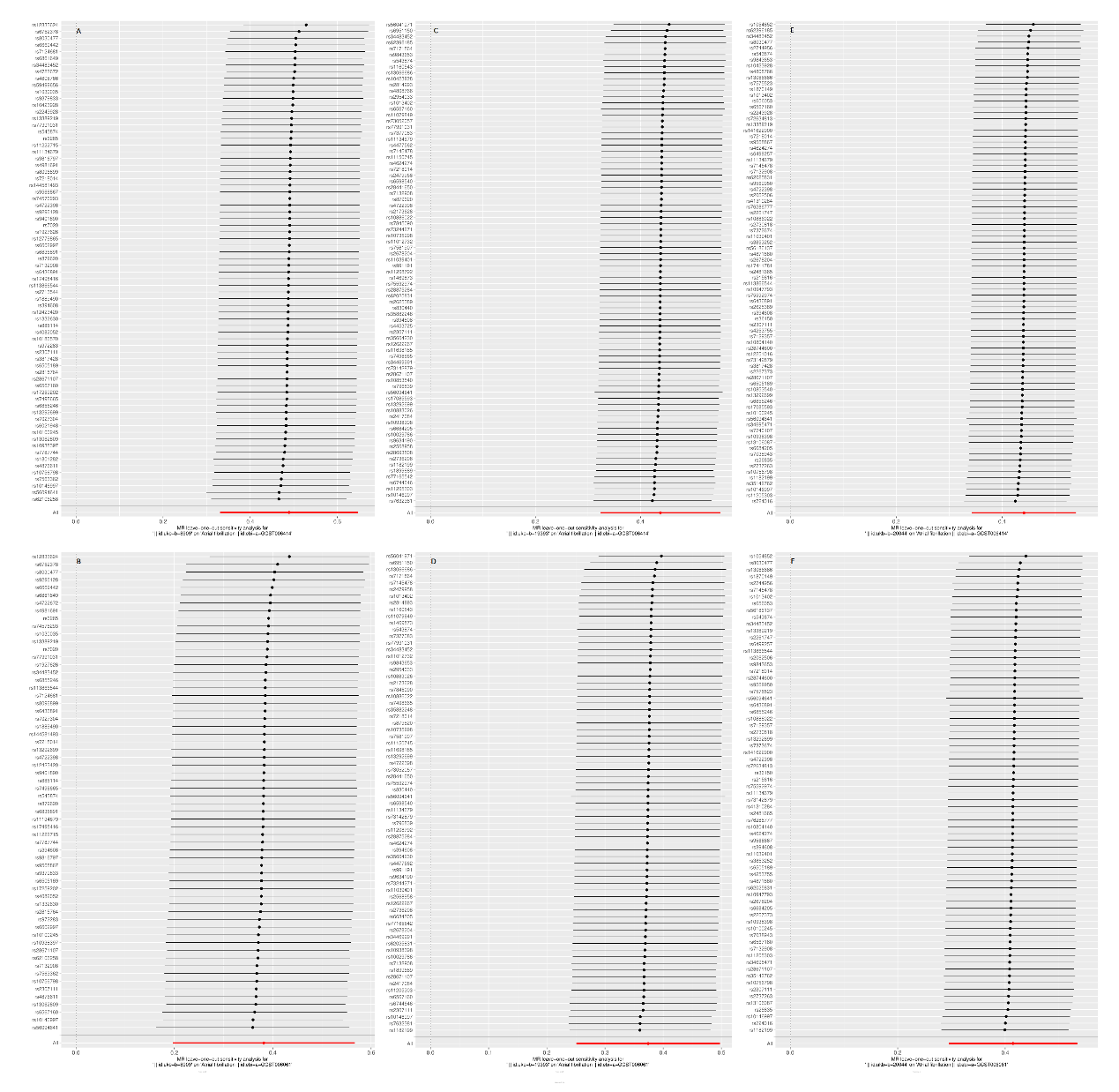


**Note:** The figure-A represents the result of the “leave-one-out” method for “Body fat percentage (ukb-b-8909)” and AF in GWAS dataset ebi-a-GCST006414, while the figure-B represents the result of the “leave-one-out” method for “Body fat percentage (ukb-b-8909)” and AF in GWAS dataset ebi-a-GCST006061.

The figure-C represents the result of the “leave-one-out” method for “Whole body fat mass (ukb-b-19393)” and AF in GWAS dataset ebi-a-GCST006414, while the figure-D represents the result of the “leave-one-out” method for “Whole body fat mass (ukb-b-19393)” and AF in GWAS dataset ebi-a-GCST006061.

The figure-E represents the result of the “leave-one-out” method for “Trunk fat mass (ukb-b-20044)” and AF in GWAS dataset ebi-a-GCST006414, while the figure-F represents the result of the “leave-one-out” method for “Trunk fat mass (ukb-b-20044)” and AF in GWAS dataset ebi-a-GCST006061.

All these results indicated that no specific SNP had a significant effect on the overall result and verified the robustness of the conclusions.
